# Knockdown of Y-box binding protein 1 induces autophagy in early porcine embryos

**DOI:** 10.3389/fcell.2023.1238546

**Published:** 2023-10-30

**Authors:** Wen-Jie Jiang, Song-Hee Lee, Geun Heo, Hak Jae Chung, Eun Seok Cho, Soo Jin Sa, Shinichi Hochi, Xiang-Shun Cui

**Affiliations:** ^1^ Department of Animal Science, Chungbuk National University, Cheongju, Republic of Korea; ^2^ Swine Science Division, National Institute of Animal Science, Cheonan-si, Republic of Korea; ^3^ Planning and Coordination Division, National Institute of Animal Science, Iseo-myeon, Republic of Korea; ^4^ Faculty of Textile Science and Technology, Shinshu University, Ueda, Japan

**Keywords:** YBX1, mitochondria, er stress, autophagy, pig

## Abstract

Y-box binding protein 1 (*YBX1*) plays important roles in RNA stabilization, translation, transcriptional regulation, and mitophagy. However, its effects on porcine preimplantation embryos remain unclear. In this study, we knocked down *YBX1* in the one-cell (1C) stage embryo via small interfering RNA microinjection to determine its function in porcine embryo development. The mRNA level of *YBX1* was found to be highly expressed at the four-cell (4C) stage in porcine embryos compared with one-cell (1C) and two-cell (2C) stages. The number of blastocysts was reduced following YBX1 knockdown. Notably, *YBX1* knockdown decreased the phosphatase and tensin homolog-induced kinase 1 (*PINK1*) and parkin RBR E3 ubiquitin protein ligase (*PRKN*) mRNA levels. *YBX1* knockdown also decreased PINK1, active mitochondria, and sirtuin 1 levels, indicating reduced mitophagy and mitochondrial biogenesis. Furthermore, *YBX1* knockdown increased the levels of glucose-regulated protein 78 (GRP78) and calnexin, leading to endoplasmic reticulum (ER) stress. Additionally, *YBX1* knockdown increased autophagy and apoptosis. In conclusion, knockdown of *YBX1* decreases mitochondrial function, while increasing ER stress and autophagy during embryonic development.

## 1 Introduction

A mitochondrion is an essential organelle that controls energy conversion and ATP production (Matsumoto et al., 2012). Mitochondrial fission and fusion maintain the mitochondrial morphology, homeostasis, and inheritance via mitochondrial biogenesis and mitophagy (Ma et al., 2020). Mitophagy is crucial for mitochondrial quality control (Dlamini et al., 2021). Dysfunctional mitochondria can generate apoptotic signals to induce cell death (Zhang, 2013). Endoplasmic reticulum (ER) is another important organelle which coordinates stress-related signaling pathways critical for maintaining the crosstalk between intracellular and extracellular environments (Lin et al., 2008; Ron and Hubbard, 2008; Kuo et al., 2022). Excess unfolded or misfolded proteins accumulate in the ER lumen leading to ER stress, disrupting ER function, thereby activating the unfolded protein response (UPR) (Senft and Ronai, 2015). ER and mitochondria together regulate various cellular processes, for example, lipid biosynthesis, apoptosis, and mitophagy (Dlamini et al., 2021).

Y-box binding protein 1 (YBX1) is a cytoplasmic messenger ribonucleoprotein that can bind to RNA (Deng et al., 2022). It modulates RNA stability, translation, and transcription (Mordovkina et al., 2020). *YBX1* is necessary for cancer cell proliferation and embryonic development. In human cervical cancer HeLa cells, *YBX1* affects mitochondrial oxidative phosphorylation proteins expression (Matsumoto et al., 2012). In goat embryos, YBX1 was studied to regulate splicing and maternal mRNA decay (Deng et al., 2022). In mouse, YBX1 is involved in early mouse development, including neural tube closure and cell proliferation (Uchiumi et al., 2006). In zebrafish, YBX1 affects oocyte maturation and maternal-to-zygotic transition process (Sun et al., 2018). Phosphatase and tensin homolog-induced kinase 1 (*PINK1*) is associated with Parkinsonian disorders (Valente et al., 2004a; Valente et al., 2004b). *PINK1* plays a key role in mitochondrial quality control via the *PINK1* and Parkin pathways (Niu et al., 2019). *YBX1* knockdown decreases the stability of PINK1 and parkin RBR E3 ubiquitin protein ligase (PRKN) to regulate mitophagy for brown adipogenesis and thermogenesis (Wu et al., 2022). *PINK1* knockdown impairs embryonic development and induces mitochondrial dysfunction, autophagy, and apoptosis (Niu et al., 2019).

In this study, we hypothesized that YBX1 is crucial for porcine embryonic development. To evaluate this hypothesis, we reduced the expression of YBX1 by microinjection of YBX1 siRNA to explore the role of YBX1 during the early development of porcine embryos. Additionally, we examined mitochondrial function, GRP78, calnexin, LC3, and caspase 3 following YBX1 knockdown. The results suggest that YBX1 affects embryonic development by influencing mitochondrial function, ER stress, autophagy, and apoptosis.

## 2 Material and methods

Unless otherwise stated, all chemicals were purchased from Sigma-Aldrich (St. Louis, MO, United States of America).

### 2.1 Oocyte collection and *in vitro* maturation

Pre-pubertal porcine ovaries were collected from a local abattoir (Farm Story Dodarm B&F, Umsung, Chungbuk, South Korea) and transported to the laboratory at 37°C in phosphate-buffered saline (PBS). Porcine cumulus–oocyte complexes (COCs) were aspirated from small antral follicles (diameter: 3–6 mm). Oocytes surrounded by intact cumulus layers were washed thrice with the *in vitro* maturation (IVM) medium (11150-059; Thermo Fisher Scientific, Waltham, MA, United States) containing TCM199, 0.1 g/L sodium pyruvate, 0.6 mM cysteine, 10 ng/mL epidermal growth factor, 10% (v/v) porcine follicular fluid, 10 IU/mL luteinizing hormone, and 10 IU/mL follicle-stimulating hormone. COCs were randomly divided and cultured in IVM medium (500 μL) for 44 h at 38.5°C under 5% CO2.

### 2.2 Parthenogenetic activation and *in vitro* culture

Briefly, 1 mg/mL hyaluronidase was used to detach the cumulus cells. Oocyte with a polar body was selected for activation. Denuded oocytes were parthenogenetically activated using two direct-current pulses of 110 V for 60 µs in 280 mM mannitol containing 0.1 mM CaCl_2_, 0.05 mM MgSO_4_, 0.01% polyvinyl alcohol (PVA, w/v), and 0.5 mM 4-(2-hydroxyethyl) piperazine-l-ethanesulfonic acid. After that, activated oocytes were cultured with 0.4% bovine serum albumin (BSA) and 7.5 μg/mL cytochalasin B in PZM-5 for 3 h to inhibit pseudo-second polar body extrusion. Activated oocytes were washed 3 times and cultured in PZM-5 with 0.4% BSA in a 4-well plate under the same condition as IVM. Subsequently, embryos were cultured for 7 days, and then examined the blastocyst development rate (blastocyst development rate = number of blastocysts/number of embryos).

### 2.3 Microinjection

For the knockdown groups, the sequences of the small interfering RNAs (siRNAs) was the same with previous study (Jiang et al., 2023b). *YBX1* siRNA (50 μM) was microinjected into the cytoplasm of a parthenogenetically activated oocyte via an Eppendorf Femto-Jet (Eppendorf, Hamburg, Germany) and Nikon Diaphot Eclipse TE300 inverted microscope (Nikon, Tokyo, Japan) equipped with the Narishige MM0-202N hydraulic 3-dimensional micromanipulator (Narishige, Amityville, NY, United States). As a control, the company provided negative siRNA (sense: UUC​UCC​GAA​CGU​GUC​ACG​UTT, antisense: ACG​UGA​CAC​GUU​CGG​AGA​ATT) was microinjected into the cytoplasm of a parthenogenetically activated oocyte under the same conditions. Embryos were cultured in PZM-5 medium for 1 or 2 or 7 d after microinjection.

### 2.4 Immunofluorescence staining

According to previous article (Niu et al., 2019), 4% paraformaldehyde in PBS was used to fixed embryos for 1 h at room temperature. Then, embryos were washed thrice and incubated with 1% Triton X-100 in PBS for 1 h at room temperature. Embryos were washed thrice and blocked with 3% BSA in PVA-PBS for 1 h. Embryos were then incubated overnight in different primary antibodies: rabbit anti-YBX1 (1:100, 20339-1-AP, Proteintech), rabbit anti-PINK1 (1:100, 23274-1-AP, Proteintech), rabbit anti-GRP78 (1:100, ab21685, Abcam), rabbit anti-calnexin (1:100, ab22595, Abcam), light chain 3 (LC3) (1:100, NB100-2220, Novus biologicals), mouse anti-TOM20 (1:50, SC-17764, Santa Cruz Biotechnology) and rabbit anti-caspase 3 (1:100, 9664S, Cell Signaling Technology) at 4°C. Following three washes (5 min each) with PVA-PBS, embryos were stained with different Alexa Fluor secondary antibodies for 1 h at room temperature. After washing 3 times, embryos were mounted on slides, and examined by confocal microscope (Zeiss LSM 710 Meta). Images were processed using the Zen software (v.8.0; Zeiss).

### 2.5 Colocalization assay of mitochondria and TOM20

For the colocalization of mitochondria and TOM20, 4-cell stage embryos were incubated with 500 nM MitoTracker Red CMXRos (M7512; Thermo Fisher Scientific) at 38.5°C for 30 min. After 3 washes with PZM-5, the staining of TOM20 was the same as in the immunofluorescence staining.

### 2.6 Protein extraction and western blotting analysis

60 embryos per group (control group and YBX1 knockdown group) were placed in 20 μL of ice-cold 1 × sodium dodecyl sulfate [SDS] sample buffer and incubated at 98°C for 10 min. Based on the previous article (Sui et al., 2020; Sun et al., 2023), the proteins in each sample were separated using 10% SDS-polyacrylamide gel electrophoresis and transferred to a polyvinylidene fluoride membrane (Millipore, Bedford, MA, United States) via electroblotting. The target protein binding site was blocked with Tris-buffered saline containing Tween-20 (TBST) and 5% skim milk powder for 1 h at room temperature. Subsequently, the membranes were incubated with different antibodies: rabbit anti-YBX1 (1:1,000, 20339-1-AP, Proteintech), rabbit anti-GRP78 (1:1,000, ab21685, Abcam), rabbit anti-calnexin (1:1,000, ab22595, Abcam), light chain 3 (LC3) (1:1,000, NB100-2220, Novus biologicals) and mouse anti-SIRT1 (1:1,000, 60303-1, Proteintech) overnight at 4°C. The membranes were washed three times with TBST for 10 min each. The membranes were then incubated in secondary antibody (1:20,000) for 1 h at room temperature. The membranes were then washed three times with TBST and exposed to the SuperSignal West Femto Maximum Sensitivity Substrate (Thermo Fisher Scientific). To quantify the Western blot results, the intensities of the bands were analyzed by the ImageJ software.

### 2.7 Reverse transcription-quantitative polymerase chain reaction (RT-qPCR)

RNA was extracted from a pool of 30 embryos per group (control group and YBX1 knockdown group) using the Dynabead mRNA DIRECT kit (61,012; Thermo Fisher Scientific). cDNA was synthesized using a cDNA synthesis kit (Thermo Fisher Scientific), according to the manufacturer’s instructions. Quantitative reverse transcription-PCR was performed using a fast real-time PCR system (ABI StepOnePlus). Real-time quantitative PCR (qPCR) was performed according to previous article (Jiang et al., 2023a). *18S* rRNA was used as the reference gene. All primer sequences used in this study are listed in Table 1. Relative genes expression levels were determined using the 2^−ΔΔCT^ method.

**TABLE 1:** Information of primers used for RT-PCR.

Genes	Primer sequences	Accession No.	Product size (bp)
*YBX1*	F: CTT​CAA​TTA​CCG​GCG​CAG​AC	XM_021096922.1	172
	R: CTT​CTT​GGT​GGA​TGA​CCG​GA		
*PINK1*	F: CCG​CAG​TTA​CCA​AGA​AGC​TC	XM_021095478.1	181
	R: TTT​CAG​GTT​CTT​CAG​GGC​CA		
*PARKIN*	F: CTC​AGG​GTC​CTT​CTT​GCT​GG	NM_001044603.2	189
	R: TGA​TGC​AGG​TGA​TGT​CTC​GG		
*Caspase 3*	F: TCT​AAC​TGG​CAA​ACC​CAA​ACT​T	NM_214131.1	85
	R: AGT​CCC​ACT​GTC​CGT​CTC​AAT		
*BAX*	F: GAA​TGG​GGG​GAG​AGA​CAC​CT	XM_013998624.2	180
	R: CCGCCACTCGGAAAAAGA		
*BCL2*	F: GAA​CTG​GGG​GAG​GAT​TGT​GG	XM_021099593.1	189
	R: CAT​CCC​AGC​CTC​CGT​TAT​CC		
*LC3*	F: CCG​AAC​CTT​CGA​ACA​GAG​AG	NM_001190290	206
	R: AGG​CTT​GGT​TAG​CAT​TGA​GC		
*18S*	F: CGC​GGT​TCT​ATT​TTG​TTG​GT	NR_046261	219
	R: AGT​CGG​CAT​CGT​TTA​TGG​TC		

Note: The annealing temperature for all reactions was 60°C.

F: forward primer; R: reverse primer.

### 2.8 Statistical analysis

Each experiment was repeated at least three times and results were presented as the mean ± standard error of the mean. The GraphPad Prism 5 software (GraphPad, San Diego, CA, United States of America) was used for statistical analysis. A *t*-test was used to compare the results between groups. *p* < 0.05 was considered statistically significant.

## 3 Results

### 3.1 Subcellular distribution and expression of YBX1 during embryo development

**FIGURE 1:**
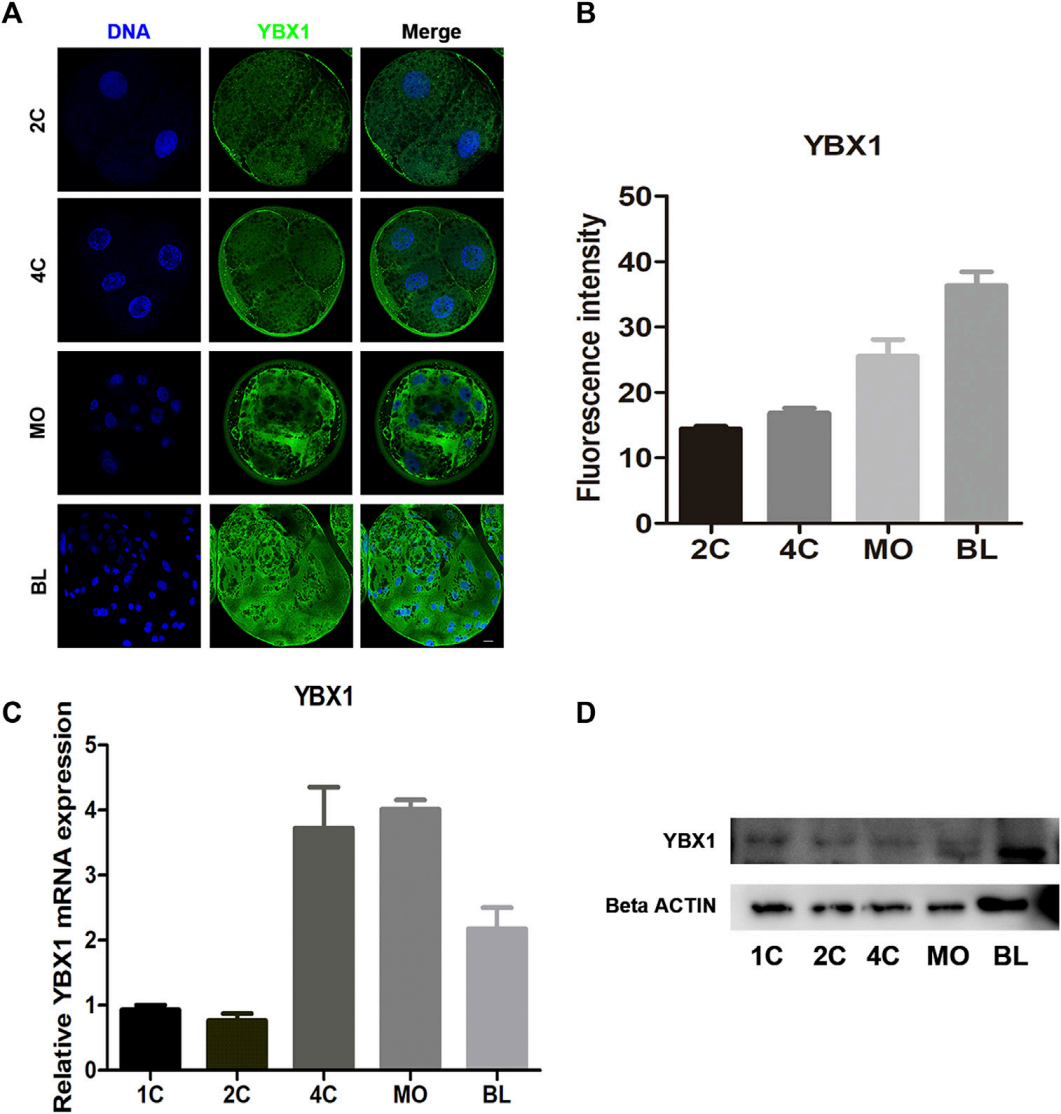
Subcellular distribution and expression of YBX1 during embryo development. **(A)** Immunofluorescence images of YBX1 at two-cell (2C), four-cell (4C), morula (MO) and blastocyst (BL) stages. Blue, DNA; green, YBX1. Scale bar, 40 µm. **(B)** Fluorescence intensity for YBX1 expression at 2C, 4C, MO, BL stages. **(C)** Real-time quantitative PCR results of *YBX1* mRNA expression levels during early porcine embryonic development. **(D)** Western blotting results of YBX1 protein expression levels during early porcine embryonic development.

To investigate its subcellular localization during embryonic development, we performed immunofluorescence staining to determine the location of YBX1 in two-cell (2C; n = 6), four-cell (4C; n = 6), morula (MO; n = 5) and blastocyst (BL; n = 6) stage embryos. As shown in Figures 1A,B, YBX1 localized in the cytoplasm and the immunofluorescence (IF) intensity of YBX1 was gradually increased. Next, we examined the mRNA levels of *YBX1* during embryonic development. We observed that the mRNA levels of *YBX1* were increased from the 4C to BL stage compared with the 1C and 2C stages (Figure 1C), indicating that YBX1 is a zygotic gene. Moreover, the results of Western blotting were similar to those of IF and real-time quantitative PCR. Taken together, these data indicate the presence of YBX1 in porcine embryos, which may play a key role in embryonic development.

### 3.2 *YBX1* knockdown impairs embryo development

**FIGURE 2:**
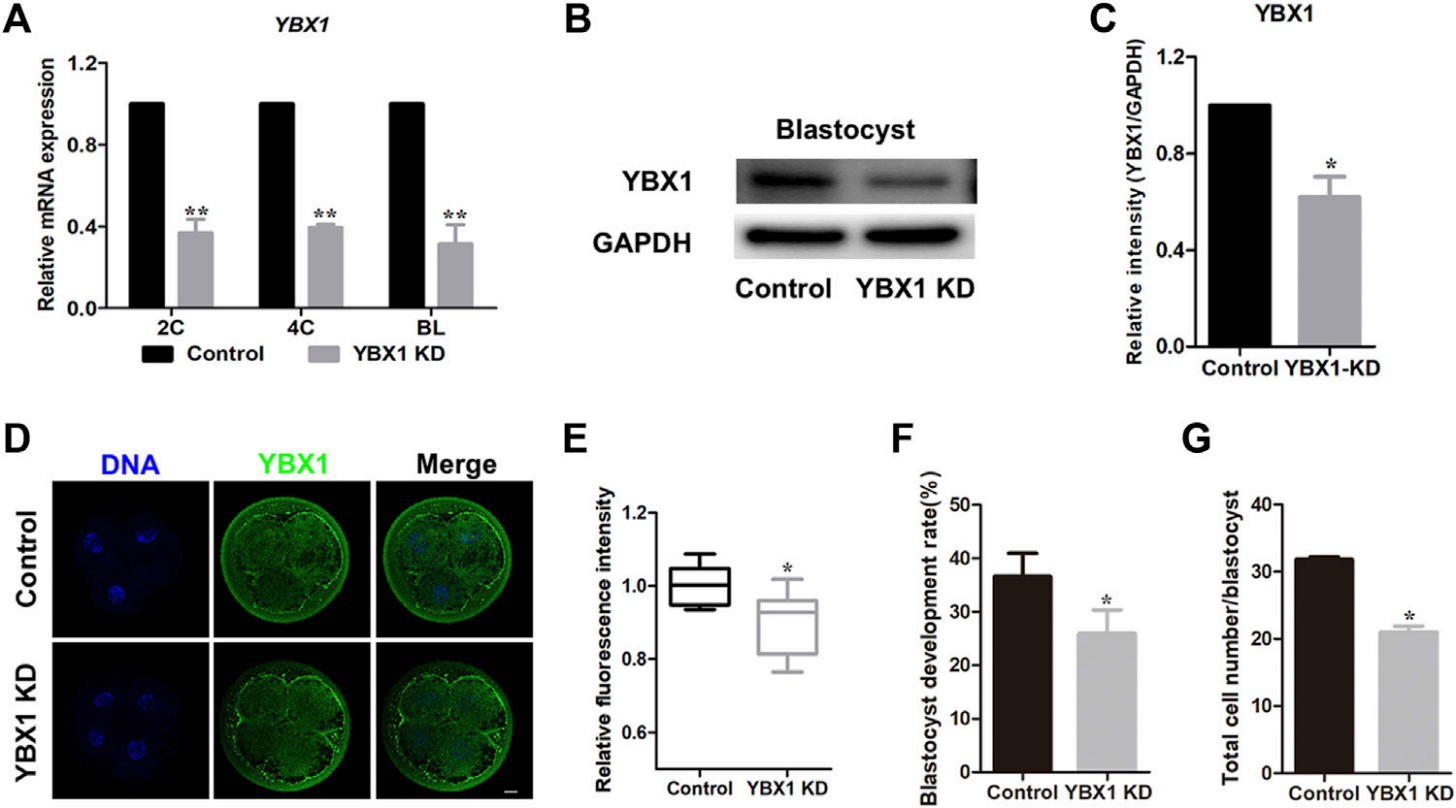
*YBX1* knockdown impairs embryo development. **(A)** Real-time quantitative PCR results of *YBX1* mRNA expression levels in the control and YBX1 KD groups. Compared with the control group, the expression level of *YBX1* mRNA was significantly lower in the YBX1 KD group. **(B)** *YBX1* knockdown was confirmed via Western blotting. **(C)** Relative YBX1 protein intensity after *YBX1* knockdown. **(D)** Immunofluorescence staining of YBX1 in the control and YBX1 KD groups at the four-cell stage. Blue, DNA; green, YBX1. Scale bar, 20 µm. **(E)** Relative fluorescence intensity of YBX1 at the four-cell stage. The relative fluorescence intensity of YBX1 was significantly lower in the YBX1 KD group at the 4C stage compared to the control group. **(F)** Blastocyst development rate after *YBX1* knockdown. **(G)** Total cell number/blastocyst after *YBX1* knockdown. *p < 0.05, **p < 0.01 indicate significant differences between groups.

To explore the functional roles of *YBX1* during embryonic development in pigs, we knocked down *YBX1* at the 1C stage. As shown in Figure 2A, mRNA levels of *YBX1* were decreased by approximately 62% in the *YBX1* knockdown group compared to those in the control group at the 2C, 4C and blastocyst stage. Knockdown of YBX1 was verified by Western blotting (0.62 ± 0.08 vs 1; *p* < 0.05; Figures 2B,C) at blastocyst stage and immunofluorescence staining (0.91 ± 0.03, n = 11 vs 1 ± 0.01, n = 12; *p* < 0.05; Figures 2D,E) at 4C stage. Moreover, blastocyst development rate (25.94 ± 4.42, n = 218 vs 36.66 ± 4.30, n = 217; *p* < 0.05; Figure 2F) and cell number in each blastocyst were decreased in the *YBX1* knockdown group than in the control group (31.86 ± 0.35, n = 31 vs 21.02 ± 0.89, n = 27; *p* < 0.05; Figure 2G). These results suggest that *YBX1* is important for embryonic development.

### 3.3 *YBX1* knockdown impairs mitochondrial function

**FIGURE 3:**
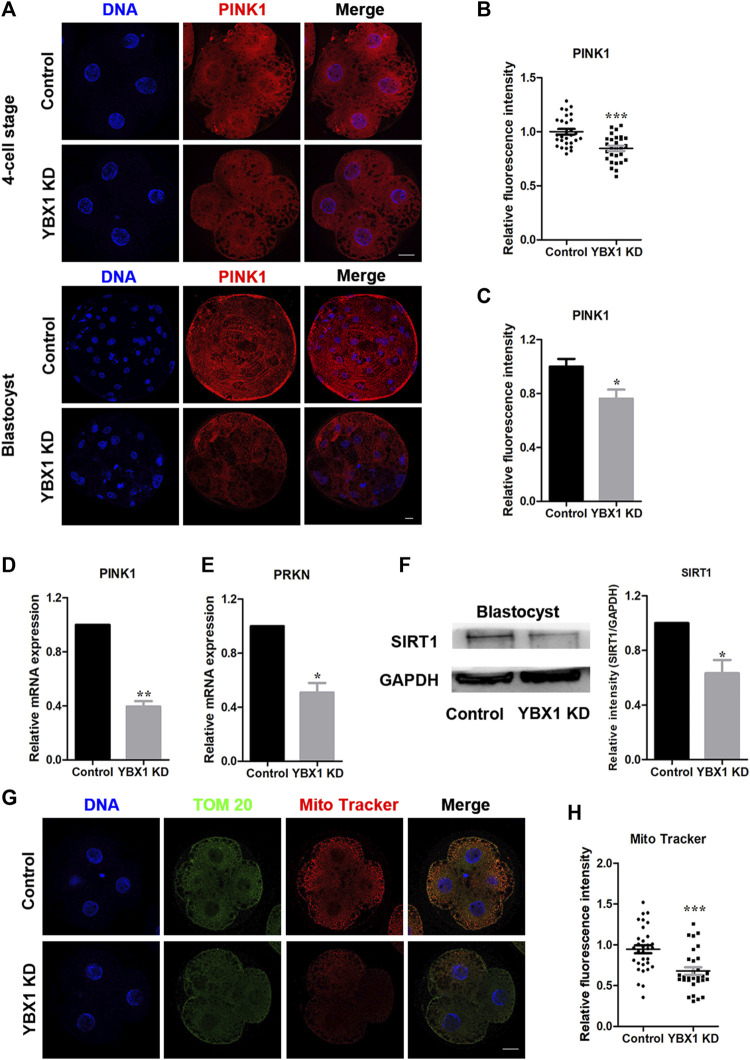
*YBX1* knockdown impairs mitochondrial function. **(A)** Immunofluorescence staining of PINK1 in the control and YBX1 KD groups at the four-cell and blastocyst stages. Blue, DNA; red, PINK1. Scale bar, 20 µm. **(B)** Relative fluorescence intensity of PINK1 at the four-cell stage. The relative fluorescence intensity of PINK1 was significantly lower in the YBX1 KD group at the 4C stage compared to the control group. **(C)** Relative fluorescence intensity of PINK1 at the blastocyst stage. The relative fluorescence intensity of YBX1 was significantly lower in the YBX1 KD group at the BL stage compared to the control group. **(D)** Relative mRNA expression of *PINK1* at the blastocyst stage. **(E)** Relative mRNA expression of PRKN at the blastocyst stage. **(F)** Protein levels of SIRT1 in the control and YBX1 KD groups. **(G)** Immunofluorescence staining of TOM20, Mito Tracker in the control and YBX1 KD groups at the four-cell stage. Blue, DNA; Green, TOM20; Red, Mito Tracker; Scale bar, 20 µm. **(H)** Relative fluorescence intensity of Mito Tracker at four-cell stage. Compared with the control group, the relative fluorescence intensity of Mito Tracker in 4C stage in the YBX1 KD group was significantly lower. *p < 0.05, **p < 0.01, ***p < 0.001 indicate significant differences between groups.

It has been reported that *YBX1* knockdown reduces the mRNA stability of two important mitophagy-associated proteins, PINK1 and PRKN (Wu et al., 2022). Therefore, we measured the expression levels of PINK1 and PRKN following *YBX1* knockdown in this study. First, we examined PINK1 expression levels in 4C and blastocyst stage embryos via fluorescent staining (Figure 3A). We found that the expression levels of PINK1 were decreased in 4C (1 ± 0.03, n = 29 vs 0.85 ± 0.02, n = 28; *p* < 0.001; Figure 3B) and blastocyst (1 ± 0.06, n = 13 vs 0.76 ± 0.07, n = 12; *p* < 0.01; Figure 3C) stage embryos. Moreover, mRNA levels of *PINK1* (1 vs 0.39 ± 0.04; *p* < 0.01; Figure 3D) and PRKN (1 vs 0.51 ± 0.07; *p* < 0.05; Figure 3E) were decreased after *YBX1* knockdown. These results suggest that *YBX1* affects the PINK1 and PRKN expression levels. Next, we investigated the influence of *YBX1* knockdown on mitochondrial biogenesis. *SIRT1* is a marker of mitochondrial biogenesis. Western blotting revealed significantly decreased SIRT1 expression after *YBX1* knockdown (1 vs 0.63 ± 0.1; *p* < 0.05; Figure 3F). Moreover, MitoTracker Red CMXRos was used to detect mitochondrial activity (Figure 3G). Mitochondrial activity was significantly decreased in YBX1 KD group (0.95 ± 0.05, n = 32 vs 0.68 ± 0.11, n = 30; *p* < 0.001; Figure 3H), these results indicate that YBX1 knockdown decreases mitochondrial function.

### 3.4 *YBX1* knockdown induces ER stress

**FIGURE 4:**
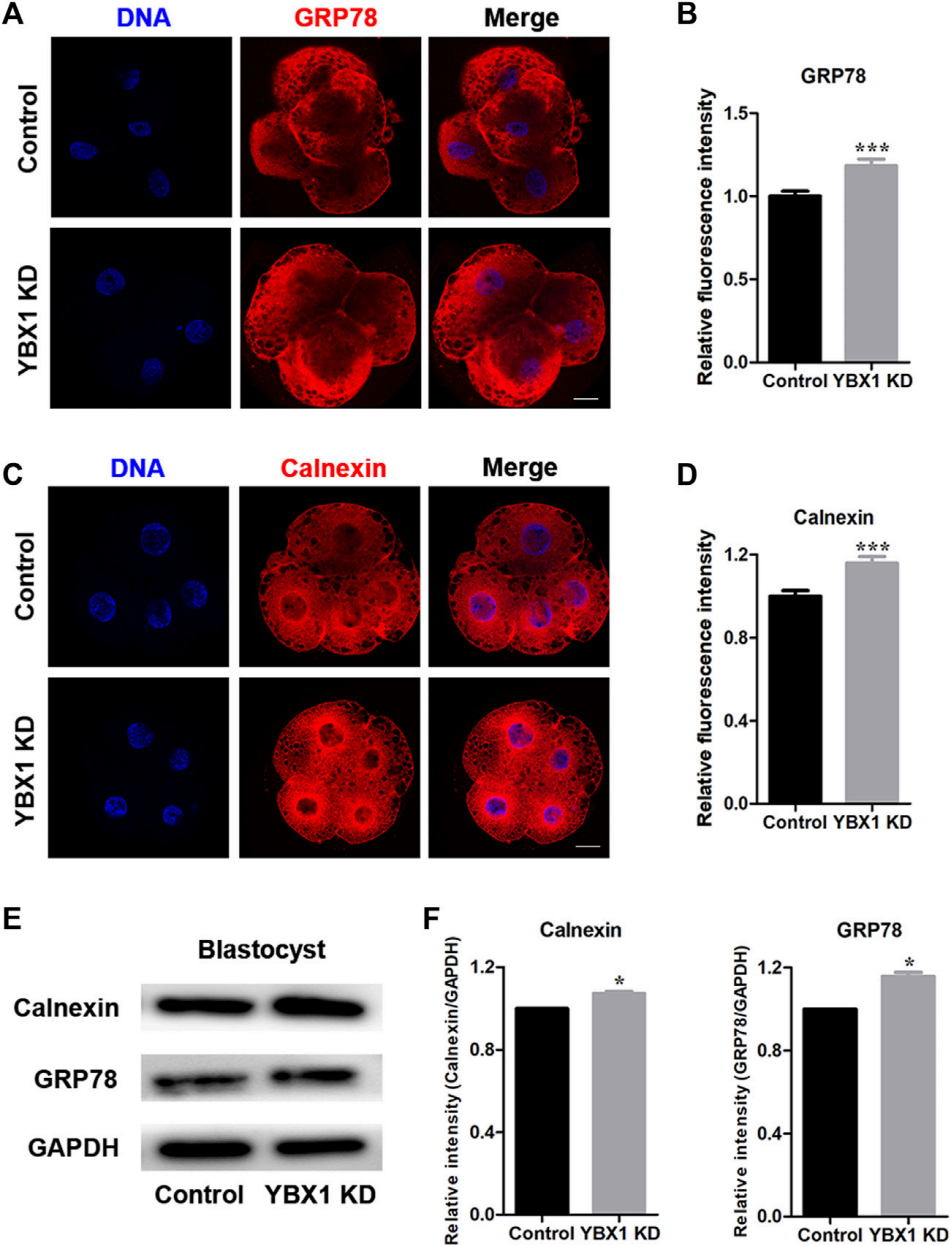
*YBX1* knockdown induces ER stress. **(A)** Immunofluorescence staining of GRP78 in the control and YBX1 KD groups at the four-cell stage. Blue, DNA; red, GRP78. Scale bar, 20 µm. **(B)** Relative fluorescence intensity of GRP78 at the four-cell stage. Compared with the control group, the relative fluorescence intensity of GRP78 in 4C stage in the YBX1 KD group was significantly higher. **(C)** Immunofluorescence staining of calnexin in the control and YBX1 KD groups at the four-cell stage. Blue, DNA; red, calnexin. Scale bar, 20 µm. **(D)** Relative fluorescence intensity of calnexin at the four-cell stage. Compared with the control group, the relative fluorescence intensity of calnexin in 4C stage in the YBX1 KD group was significantly higher. **(E)** Protein levels of calnexin and GRP78 in the control and YBX1 KD groups. **(F)** Relative protein levels of calnexin and GRP78. *p < 0.05 and ***p < 0.001 indicate significant differences between groups.

Next, we investigated the association of *YBX1* knockdown with ER stress and determined the ER stress-related proteins expression, calnexin and GRP78 (Figure 4 A and C). As shown in Figures 4B,D, expression levels of GRP78 (1 ± 0.03, n = 23 vs 1.18 ± 0.04, n = 22; *p* < 0.001) and calnexin (1 ± 0.03, n = 21 vs 1.16 ± 0.03, n = 21; *p* < 0.001) were all significantly increased after *YBX1* knockdown. Meanwhile, protein expression levels of BiP/GRP78 (1 vs 1.16 ± 0.02; *p* < 0.05; Figures 4E,F) and calnexin (1 vs 1.07 ± 0.01; *p* < 0.05; Figures 4E,F) were significantly increased after *YBX1* knockdown. Taken together, these results indicate that *YBX1* knockdown induces ER stress during embryonic development.

### 3.5 *YBX1* knockdown induces autophagy and apoptosis

**FIGURE 5:**
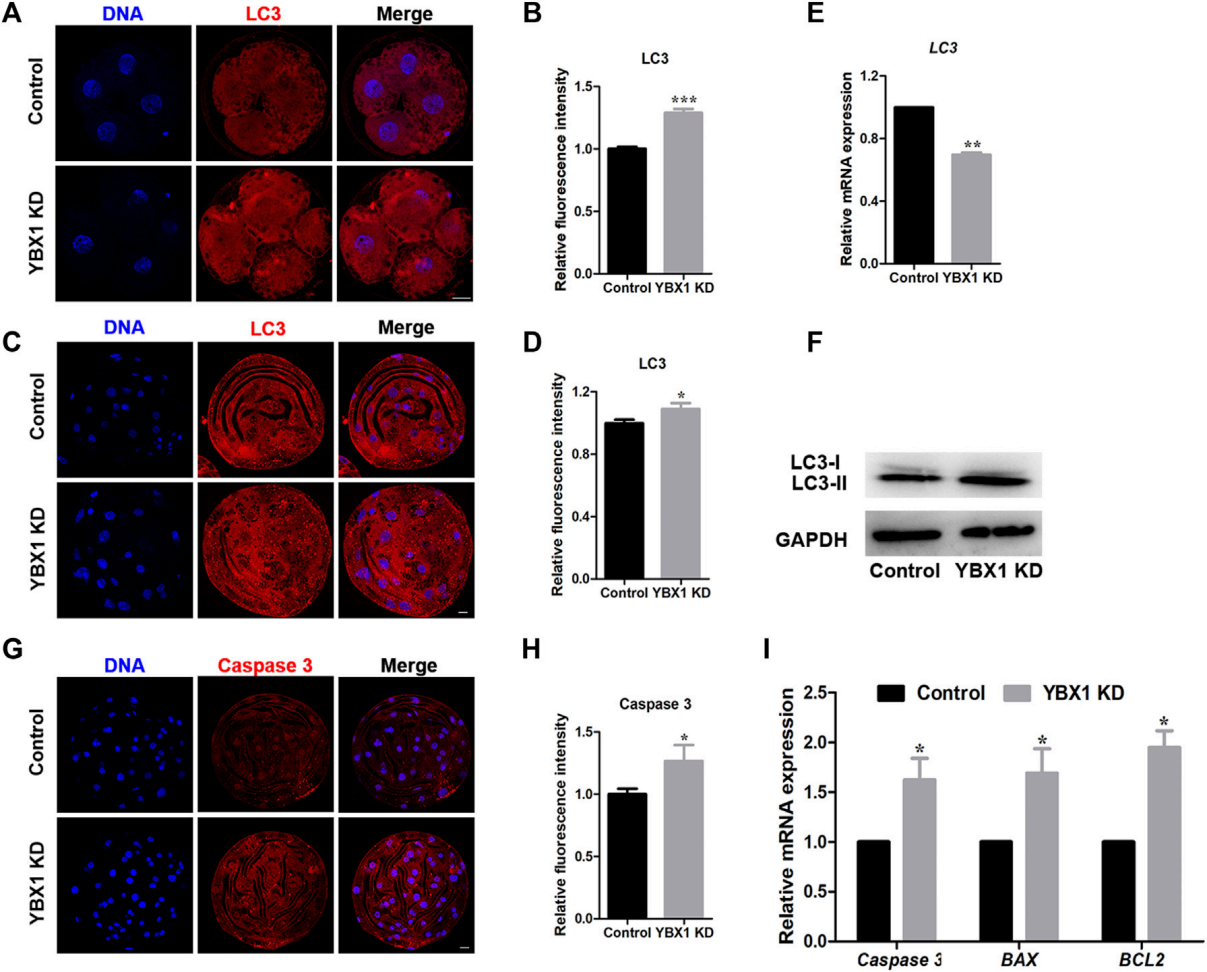
*YBX1* knockdown induces autophagy and apoptosis. **(A)** Immunofluorescence staining of LC3 in the control and YBX1 KD groups at the four-cell stage. Blue, DNA; red, LC3. Scale bar, 20 µm. **(B)** Relative fluorescence intensity of LC3 at the four-cell stage. Compared with the control group, the relative fluorescence intensity of LC3 in 4C stage in the YBX1 KD group was significantly higher. **(C)** Immunofluorescence staining of LC3 in the control and YBX1 KD groups at the blastocyst stage. Blue, DNA; red, LC3. Scale bar, 20 µm. **(D)** Relative fluorescence intensity of LC3 at the blastocyst stage. Compared with the control group, the relative fluorescence intensity of LC3 in BL stage in the YBX1 KD group was significantly higher. **(E)** Relative mRNA expression of LC3 at the four-cell stage. **(F)** Relative protein levels of LC3 in the control and YBX1 KD groups. **(G)** Immunofluorescence staining of caspase 3 in the control and YBX1 KD groups at the blastocyst stage. Blue, DNA; red, caspase 3. Scale bar, 20 µm. **(H)** Relative fluorescence intensity of caspase 3. Compared with the control group, the relative fluorescence intensity of Caspase 3 in blastocyst stage in the YBX1 KD group was significantly higher. **(I)** Relative mRNA expression of *Caspase3*, *BAX* and *BCL2* at the four-cell stage. *p < 0.05, **p < 0.01 and ***p < 0.001 indicate significant differences between groups.

Autophagy and apoptosis are important for maintaining organismal and cellular homeostasis (Fan and Zong, 2013). As mitochondrial and ER impairment can induce autophagy and apoptosis, we first evaluated the influence of *YBX1* knockdown on autophagy. As shown in Figures 5A–D, LC3 levels were significantly increased in 4C (1 ± 0.02, n = 25 vs 1.29 ± 0.03, n = 19; *p* < 0.001) and blastocyst (1 ± 0.02, n = 21 vs 1.09 ± 0.04, n = 19; *p* < 0.05) stage embryos of the *YBX1* knockdown group compared to the control group. This result was confirmed by Western blotting (1 vs 1.29 ± 0.03; *p* < 0.05; Figure 5F), although the mRNA level of LC3 decreased (1 vs 0.70 ± 0.01; *p* < 0.01; Figure 5E). Therefore, autophagy was observed in *YBX1* knocked down porcine embryos. Next, we assessed cell apoptosis via immunofluorescence staining for caspase 3. As shown in Figures 5G,H, staining intensity of caspase 3 was significantly increased in YBX1 knockdown group than that in the control group (1 ± 0.04, n = 18 vs 1.27 ± 0.13, n = 13; *p* < 0.05). Moreover, the apoptosis related genes *Caspase 3* (1 vs 1.62 ± 0.2; *p* < 0.05; Figure 5I), *BAX* (1 vs 1.69 ± 0.24; *p* < 0.05; Figure 5I) and *BCL2* (1 vs 1.95 ± 0.17; *p* < 0.05; Figure 5I) were significantly increased. According to the above results, *YBX1* knockdown induces autophagy and apoptosis.

## 4 Discussion

**FIGURE 6:**
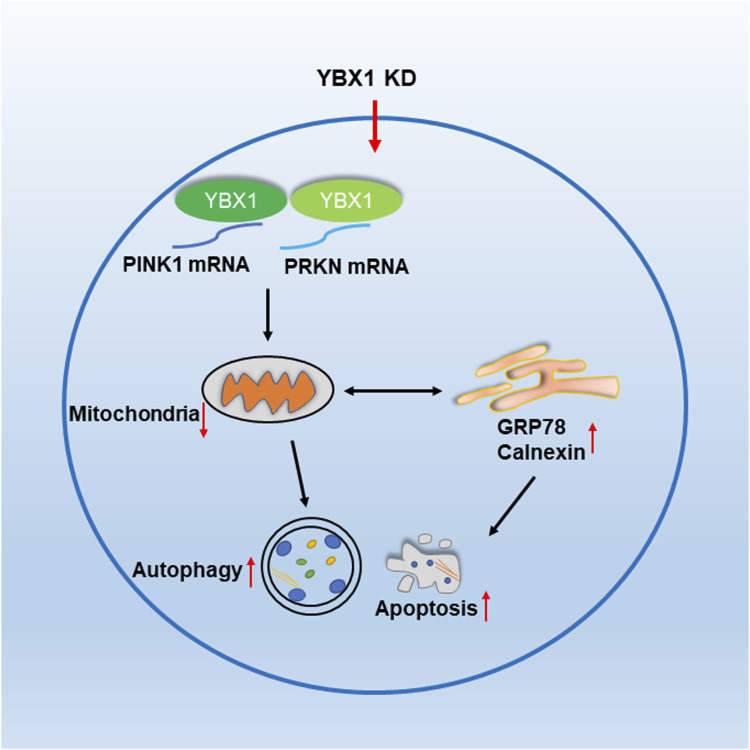
*YBX1* is important for embryonic development in pigs. *YBX1* knockdown decreases PINK1 and PRKN levels and affects mitochondrial function, thereby inducing ER stress, autophagy, and apoptosis.

In this study, we found that the loss of *YBX1* decreased PINK1 and PRKN expression levels, thereby decreasing mitochondrial function and increasing ER stress autophagy as well as apoptosis during embryonic development (Figure 6).

Mitophagy is the selective degradation of mitochondria via autophagy (Dlamini et al., 2021). It usually occurs in mitochondria that are defective after injury or stress. Mitophagy promotes the renewal of mitochondria and prevents the accumulation of dysfunctional mitochondria, which may lead to cellular degeneration (Wang et al., 2022). Mitophagy is activated in mammalian cells by PINK1/Parkin-mediated mitophagy (Iorio et al., 2021). PINK1 affects the health of mitochondrial (Nguyen et al., 2016). PINK1 activates Parkin via phosphorylation, which is an E3 ubiquitin ligase located in the cytoplasm (Bingol and Sheng, 2016; Wang et al., 2022). YBX1, a positive regulator of mitophagy, by enhancing PINK1/PRKN-mediating mitophagy in brown adipocyte (Wu et al., 2022). In this study, *YBX1* knockdown decreased *PINK1* and *PRKN* mRNA levels and reduced PINK1 protein levels, indicating that *YBX1* may regulate their mRNA stability and protein expression to influence mitophagy. SIRT1 is a marker of mitochondrial biogenesis (Majeed et al., 2021). Previous studies have shown that SIRT1 has emerged as an important regulator of mitochondrial function (Jian et al., 2012; Li et al., 2017; Niu et al., 2020). MitoTracker Red CMXRos was used to detect mitochondrial activity. It has been shown that decreased mitochondrial activity decreases mitochondrial function (Niu et al., 2019; Niu et al., 2020). Additionally, in aged oocytes, decreased MitoTracker Red were accompanied by decreased PINK1 and PRKN (Niu et al., 2020). Here, knockdown of *YBX1* significantly reduced *SIRT1* expression and mitochondrial activity, indicating that *YBX1* is important for mitochondrial biogenesis. Therefore, *YBX1* is crucial for mitophagy and mitochondrial biogenesis.

ER serves as a crucial organelle involved in the biosynthesis of lipids, proteins, and secreted proteins as well as an important site of calcium homeostasis (Lin et al., 2019). ER stress is triggered by the accumulation of unfolded or misfolded proteins in the ER that induce UPR (Feldman et al., 2005). UPR is mainly regulated by three sensors, including BiP (also known as GRP78) (Ghemrawi et al., 2018; Song et al., 2018). ER–mitochondrial play important roles in regulating the mitochondrial dynamics, inflammation, autophagy, and apoptosis. Therefore, we examined ER function in porcine embryos. The results indicated that *YBX1* knockdown increased the expression levels of calnexin and GRP78, inducing ER stress. Subsequently, increased ER stress induced UPR. Previous article indicated that *YBX1* depletion induces UPR (Sun et al., 2018), which is consistent with our finding.

Autophagy is a crucial cellular response to stress that degrades and removes unfolded proteins and damaged organelles to protect the cells (Yu et al., 2004; Dikic and Elazar, 2018). As mitophagy and ER stress induce autophagy and apoptosis, we determined the expression levels of their markers, LC3 and caspase 3 during embryonic development. We found that *YBX1* knockdown increased LC3 protein level. In cancer cells and preadipocytes, YBX1 knockdown reduces LC3 protein expression (Wu et al., 2022; Gong and Zhang, 2023; Wu et al., 2023). In this study, LC3 mRNA levels were decreased but protein expression was increased, contrary to the results, which may be due to the uniqueness of different cells or tissues. Additionally, YBX1 has been identified as an RNA-binding protein and a DNA-binding protein, mainly involved in translational repression, RNA stabilization, and transcriptional regulation. One study showed that overall translation levels were increased in YBX1-depleted embryos (Sun et al., 2018), which may lead to increased LC3 protein levels. GRP78 is important for both ER stress and autophagy (Li et al., 2008). *PINK1* knockdown increases autophagy and apoptosis (Niu et al., 2019). Here, *YBX1* knockdown increased GRP78 level and decreased PINK1 level, thereby increasing autophagy. In addition, *YBX1* knockdown has been reported to upregulate the levels of apoptosis-related genes, such as *FAS*, tumor necrosis factor, and caspase (Kloetgen et al., 2020). One study reported that oxygen-glucose deprivation/reoxygenation downregulates *YBX1* expression, whereas *YBX1* overexpression attenuates growth inhibition and apoptosis in PC12 cells (Tuerxun et al., 2021). However, the mRNA levels of BCL2 were elevated after YBX1 knockdown, contrary to the study by Feng et al. (Feng et al., 2021). The elevation of BCL2 may be due to cellular self-protection, which inhibits apoptosis and autophagy. BCL-x1 is a member of the Bcl-2 family of proteins, which are anti-apoptotic proteins. A previous study showed that apoptosis was increased but the mRNA levels of BCL-x1, BAX, and caspase-3 were decreased (Zhang et al., 2020). In addition, it was found that Bcl-2 and Bcl-xL enhance autophagy under certain conditions, such as in response to treatment with etoposide and staurosporine (Fan and Zong, 2013). Moreover, YBX1 is a DNA/RNA-binding protein that affects transcription and translation. The levels of transcription and translation are not exactly the same; therefore, an increase in BCL2 mRNA level does not mean that the protein is also elevated. Therefore, it is possible that BCL2 is increased in YBX1 knockdown embryos. These findings indicate that *YBX1* knockdown induces autophagy and apoptosis.

Taken together, our results indicate that *YBX1* decreases PINK1 and PRKN expression levels and mitochondrial function and induces ER stress, thereby causing autophagy and apoptosis during embryonic development.

## Data Availability

The original contributions presented in the study are included in the article/supplementary material, further inquiries can be directed to the corresponding author.
